# Objective and Subjective Evaluation of Saccadic Eye Movements in Healthy Children and Children with Neurodevelopmental Disorders: A Pilot Study

**DOI:** 10.3390/vision5020028

**Published:** 2021-06-07

**Authors:** Carmen Bilbao, David P. Piñero

**Affiliations:** 1 Department of Optometry, Policlínica Alto Aragón, 22003 Huesca, Spain; librabil@hotmail.com; 2 Group of Optics and Visual Perception, Department of Optics, Pharmacology and Anatomy, University of Alicante, 03690 San Vicente de Raspeig-Alicante, Spain; 3 Department of Ophtalmology, Vithas Medimar International Hospital, 03016 Alicante, Spain

**Keywords:** saccades, neurodevelopmental disorders, eye tracker, saccadic movements, dyslexia, attention deficit/hyperactivity disorder, developmental coordination disorder, oculomotricity

## Abstract

The objective of this study was to characterize saccades in children with neurodevelopmental disorders (NDDG, 17 children, age: 7–12 years) and compare them with a control group (CG, 15 children, age: 7–12 years), comparing the outcomes obtained with a subjective score system (Northeastern State University College of Optometry’s Oculomotor test, NSUCO) with the objective analysis obtained through a commercially available Eye Tracker (Tobii Eye X, Tobii, Stockholm, Sweden) and a specialized software analysis (Thomson Software Solutions, Welham Green, UK). Children from the NDDG obtained significantly lower NSUCO scores (*p* < 0.001) compared with CG. Concerning eye tracking analyses, we found a significantly higher number of hypometric saccades in NDGG (*p* ≤ 0.044). Likewise, we found a significantly higher percentage of regressions in the NDDG for a time interval of presentation of stimuli of 1 s (*p* = 0.012). Significant correlations were found between different NSUCO scores and percentage of regressions, number of saccades completed and number of hypometric saccades. The presence of hypometric saccades and regressions seems to be a differential characteristic sign of children with neurodevelopmental disorders that can be detected using an objective eye tracking analysis, but also using the subjective test NSUCO that can be easily implemented in all clinical settings.

## 1. Introduction

Oculomotricity is the ability of everyone to move the eyes naturally in a simple, coordinated and smooth mode while maintaining a clear, fused and fixed image at the central point of the retina. This ability has been studied since the early 1900s [1]. These eye movements must be integrated, being possible to perform them effectively and automatically with the involvement of the frontal and lower brain areas, but without the need of involving the frontal lobe, and thus allowing it to manage the functions of learning, attention, understanding, etc. [2]

Oculomotor skills have been used to predict the actions of objects and other people to anticipate an action as protection or as a reaction. In fastball sports, athletes must anticipate their opponent’s task in a noticeably short time [3]. Two main types of eye movements are commonly used to characterize the oculomotricity: saccades and smooth pursuits. Saccades are rapid movements of the eyes to make a fixation and bring the object point to the center of the retina, being the fastest type of movement that the human body can generate [4], with a mean speed of 100 to 800° per second and a frequency of 100,000 saccades per day [5]. They are crucial in the development of reading and sports [6], with a potential negative impact on them when an oculomotor problem is present. Besides this, the oculomotor performance contributes to the organization of a great variety of everyday activities, such as walking, driving, or cooking [7]. Specifically, each of these task activities has the requirement of a characteristic but flexible pattern of eye movements [7]. Therefore, the evaluation of oculomotricity, including specifically the analysis of saccadic movements, should be considered as a necessary evaluation in ocular exams in children. Thus, any oculomotor alteration that could interfere with the activities involved in the development process can be detected and treated. In addition, the analysis of oculomotricity has been even suggested to be as an additional tool helping in the diagnosis of some neurodevelopmental disorders [8].

Several studies have demonstrated that oculomotricity can be limited or altered in different health conditions, including neurological [9] or neurodevelopmental disorders (NDD), such as dyslexia, attention deficit disorder (ADHD) or developmental coordination disorder (DCD) [10]. In general, problems of ability and precision of saccades have been detected in these three NDDs, with movements of body and head associated [10,11]. Specifically, worse binocular coordination during and after the saccade, but without a stereotyped pattern of disconjugacy (divergence during the saccade and convergence after the saccade), has been reported in children with dyslexia [12]. In children with ADHD, deficits in oculomotor response inhibition have been detected [13]. Deficits in maintaining engagement in fixation and pursuit tasks have been found in children with DCD, with the presence of more anti-saccade errors [14].

The objective of this study was to characterize saccades in children with neurodevelopmental disorders and compare them with a control group, comparing the outcomes obtained with a subjective score system with the objective analysis obtained through a commercially available Eye Tracker.

## 2. Materials and Methods

### 2.1. Patients

This was a prospective non-randomized comparative study evaluating a total of 32 children with ages ranging from 6 to 12 years old at the Department of Optometry of the Policlínica Alto Aragón (Huesca, Spain). The research adhered to the principles of the Declaration of Helsinki and was approved by the ethics committee of the University of Alicante (exp. UA-2018-02).

Written informed consent was obtained from the parents of the children after they were explained the study protocol, risks and benefits. The recruitment of patients was performed in the Optometry Unit of Policlinica Alto Aragón, which is specialized in children’s vision. Children are normally referred by the Department of Ophthalmology to this unit when the parents refer to learning problems to undergo a complete visual performance examination, including analysis of accommodative, binocular and oculomotor functions. After this, each child is evaluated by the neuropediatrician to rule out the presence of any neurodevelopmental disorder. From the patients recruited, two groups were differentiated according to the following characteristics:

- Control group (CG): included 15 healthy children aged 7 to 12 years old. The inclusion criteria for this group were emmetropic ametropic (corrected with spectacles) or emmetropic children achieving a corrected distance visual acuity (CDVA) of 0.00 logMAR (20/20 Snellen) or better. Exclusion criteria were any ocular or systemic disease active at the time of examination as well as any previous ocular surgery. This group was examined by the neuropediatrician to rule out the presence of any neurodevelopmental disorder.
- Group of children diagnosed with a neurodevelopmental disorder (NDDG): included 17 children aged 6 to 10 years. Specifically, there were 7 children with dyslexia (41.1%), 6 (35.3%) with developmental coordination disorder (DCD) and 4 (23.5%) with attention deficit disorder (ADHD). A speech therapist, a psychologist and a pediatrician evaluated all of these children and made a diagnosis of NDD according to the DSM-5 criteria [15]. According to previous scientific studies, oculomotor abnormalities are expected to be present in these children [10,11,12,13,14]. No comorbidity of neurodevelopmental disorders was present in any case. The selection of these disorders was based on the type of patients attending our clinic, these disorders being the most commonly explored. Likewise, the evaluation of oculomotricity is more feasible in these children compared to other NDDs, most of them being cooperative patients.

### 2.2. Examination Protocol

A complete visual examination was performed before the analysis of oculomotricity, including the measurement of manifest and cycloplegic refraction, uncorrected (UDVA) and corrected distance visual acuity (CDVA), heterophoria with the cover test at distance and near (40 cm), near point of convergence (NPC) and oculomotor dominance.

The subjective evaluation of oculomotricity was performed with the NSUCO (Northeastern State University College of Optometry’s Oculomotor) test, which is a standardized procedure with scoring criteria [16]. The test subjectively assesses saccades by considering four performance areas that are classified into:

- Ability. Can the individual take the assigned test?
- Accuracy. What is the quality of execution?
- The level of head movement the patient uses to perform the task. Is head movement spontaneous when doing the task?
- The level of body movement used.

The protocol consists of rating each area of analysis from one to five, with five being the most optimal performance. It should be considered that attention is a factor that is clearly involved in the skills evaluated by the test, with the requirement of maintaining the attention focus as much as possible during the entire sequence. 

The test was conducted at 40 cm binocularly, with the patient sitting in front of the examiner. As fixation stimuli, small colored spheres of 0.5 cm in diameter and mounted on a rod were used. Saccades were evaluated by asking the patient to alternate fixation between two stimuli separated horizontally 20 cm [16,17]. According to the examiner’s observation, the scoring was determined according to the criteria detailed in Table 1 [16,17].

Besides this subjective evaluation, an objective evaluation of oculomotricity was carried out with the Eye Tracker Tobii Eye X (Tobii, Stockholm, Sweden) and the Clinical Eye Tracker software (Thomson Software Solutions, Welham Green, UK). For the measurement, the patient was seated at 50 cm from a 24-inch monitor where the Tobii Eye X ocular tracking bar was located. This screen was connected to the computer, where the measurement analysis was performed with the clinical Eye Tracker software. Two different tests were carried out. First, two spheres of 0.5 cm in diameter appearing consecutively every 0.5 s distanced 20 cm from each other were used to test saccades like those carried out with the NSUCO test. The second test was like the first, but with the spheres appearing every 1 s. With these tests, the examiner can determine if the child is able to find and reach the stimuli, if the saccades associated are hypo- or hypermetric and the number of regressions needed to reach the stimulus. Specifically, the following variables were measured and recorded: number of saccades or cycles performed, number of regressions (setbacks against the direction of the saccade), number of saccades completed, percentage of saccades completed, number of hypometric saccades, number of hypermetric saccades and percentage of hypometric and hypermetric saccades with respect to the completed saccades.

### 2.3. Statistical Analysis

The statistical data analysis was performed using the software SPSS v. 22.0 for Windows (IBM, Armonk, NY, USA). The Kolmogorov–Smirnov test confirmed that almost all samples did not follow a normal distribution and therefore non-parametric tests were used. The Mann–Whitney test was used to analyze the significance of differences in a great variety of clinical variables between the two groups involved in the study. The Chi-square test was used to assess the significance of the difference in the distribution of the two groups involved in the study in terms of gender and oscular dominance. The level of correlation between objective and subjective oculomotor parameters was analyzed by calculating the Spearman correlation coefficient. Likewise, the linear mathematical equation relating those objective and subjective parameters significantly correlated was calculated by means of linear regression analysis. All statistical tests were 2-tailed, and *p*-values less than 0.05 were considered statistically significant.

## 3. Results

### 3.1. General Clinical Data

A total of 32 children (13 girls and 19 boys) with a mean age of 8.8 years old (range, 6 to 12 years) were enrolled, with two groups differentiated: CG (15 children) and NDDG (17 children), with no significant differences between groups in gender (*p* = 0.555) and motor ocular dominance distribution (*p* = 0.385). Table 2 summarizes the main characteristics of these two groups and indicates which parameters showed statistically significant differences between groups. As shown, no statistically significant differences among groups were found in any parameter evaluated, although a non-significant trend to more exophoria at near and a further NPC was observed in NDDG (*p* ≥ 0.526) (Table 2).

### 3.2. Oculomotor Analysis

**Figure 1:**
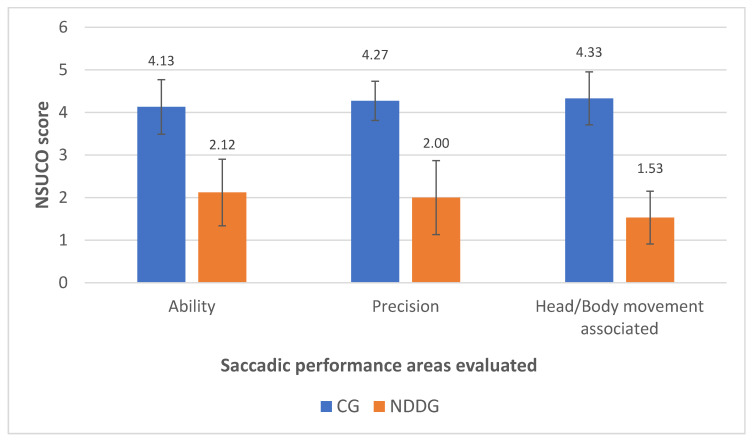
Diagram showing the statistically significant differences found between control group (CG) and neurodevelopmental disorder group (NDDG) in all saccadic performance areas evaluated with the NSUCO test.

Table 3 summarizes the results of the saccadic eye movement analysis with the NSUCO test and the eye tracker. Significant differences were found between groups in the three aspects of saccades evaluated with the NSUCO test (*p* < 0.001), with children from the NDDG obtaining lower scores (Figure 1).

**Figure 2:**
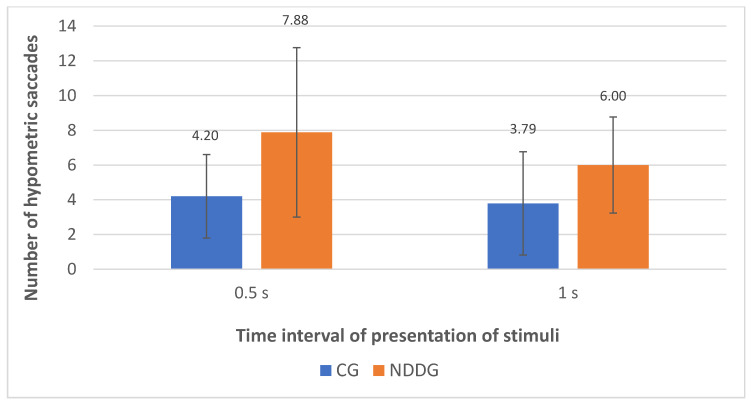
Diagram showing the statistically significant differences found between control group (CG) and neurodevelopmental disorder group (NDDG) in the number of hypometric saccades detected with the eye tracker for the two time intervals of presentation of stimuli used.

**Figure 3:**
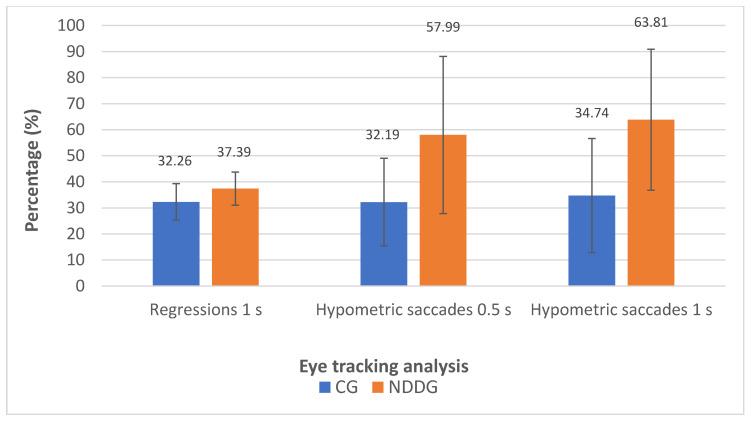
Diagram showing the statistically significant differences found between control group (CG) and neurodevelopmental disorder group (NDDG) in the percentage of regression detected with the eye tracker using a time interval of presentation of stimuli of 1 s as well as in the percentage of hypometric saccades detected using time intervals of 0.5 and 1 s.

The analysis with the eye tracker revealed the presence of statistically significant differences among groups in the number of hypometric saccades (Figure 2) as well as in the percentage of hypometric saccades with respect to the number of completed saccades for the two time intervals of presentation of stimuli used in the evaluation, 0.5 and 1 s (*p* ≤ 0.044) (Figure 3). Specifically, more hypometric saccades were observed in the NDDG compared to CG, with higher percentage of this type of anomalous movement with respect to the total number of saccades completed. Furthermore, the NDDG presented a significantly higher percentage of regressions compared to CG when the time interval of presentation of stimuli was 1 s (*p* = 0.012) (Figure 3). However, this difference did not reach statistical significance for the time interval between stimuli of 0.5 s (*p* = 0.526).

### 3.3. Correlation between Subjective and Objective Oculomotor Analysis

Several statistically significant correlations were found between the outcomes obtained with the NSUCO test and the eye tracker. The percentage of regressions for a time interval between stimuli of 1 s measured with the eye tracker correlated significantly with the scoring of three categories of the NSUCO test (ability: *r* = −0.416, *p* = 0.028; precision: *r* = −0.467, *p* = 0.012; movement body/head associated: *r* = −0.465, *p* = 0.013). Likewise, significant correlations were found between the number of saccades completed for a time interval between stimuli of 0.5 s and the NSUCO ability (*r* = 0.467, *p* = 0.012) and precision scores (*r* = 0.442, *p* = 0.019). A similar finding was obtained for the percentage of saccades completed using a time interval between stimuli of 1 s (ability: *r* = 0.489, *p* = 0.008; precision: *r* = 0.449, *p* = 0.017).

**Figure 4:**
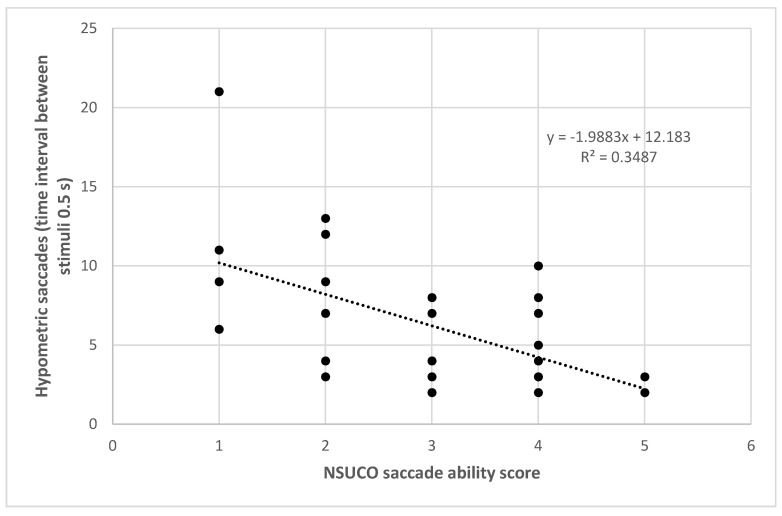
Scatter plot showing the relationship between number of hypometric saccades detected with the eye tracker using a time interval between stimuli de 0.5 s and the NSUCO saccade ability score. The adjusting lines to the data obtained by means of the least-squares fit are shown.

The number of hypometric saccades for the time interval between stimuli of 0.5 s also correlated significantly with the NSUCO ability (*r* = −0.575, *p* = 0.001) (Figure 4), precision (*r* = −0.536, p = 0.002) and movement body/head associated scores (*r* = −0.398, *p* = 0.024). The percentage of hypometric saccades with respect to the total number of saccades completed using as time interval between stimuli 0.5 s correlated significantly with the three NSUCO scorings: ability (*r* = −0.491, *p* = 0.004), precision (*r* = −0.452, *p* = 0.009), and movement of body/head associated (*r* = −0.379, *p* = 0.033). Stronger correlations were even found between the same variables but using a time interval between stimuli of 1 s in the eye tracker: ability (*r* = −0.495, *p* = 0.007), precision (*r* = −0.563, *p* = 0.002), and movement of body/head associated (*r* = −0.494, *p* = 0.008). Finally, Table 4 summarizes the results of linear regression analysis for all these significant relationships detected.

## 4. Discussion

Objective eye movement analysis by means of videoculography or any type of eye tracking has been used in different studies to characterize the oculomotor alterations in children with neurodevelopmental disorders [18]. However, the diagnostic criteria defined for the diagnosis of these conditions are not consistent, with no normative data available to be used for diagnostic purposes [18]. Different authors [10,11,12,13,14,19,20] have demonstrated the presence of oculomotor anomalies in children with neurodevelopmental disorders, but using different types of tests, parameters and cutoff criteria, being quite difficult a generalization of the diagnostic criteria and how to use this information to analyze the impact of oculomotor anomalies in daily activities, such as reading or playing sports. Furthermore, the scientific evidence of the correlation between subjective and objective tests to analyze oculomotor performance is scarce and showing poor correlation between both types of examination [21]. For example, the Developmental Eye Movement (DEM) test performance was found to be poorly correlated with saccadic eye movement skills evaluated by means of an eye tracker or symptomatology [21]. In the current study, the NSUCO test was used as subjective method that allows the clinician to evaluate the ability, precision and associated head or body movements when performing saccades. This test has been shown to be useful to detect oculomotor alterations in children with sensory processing disorders [22]. To this date, there are no studies analyzing the correlation of the outcomes obtained with this text with those obtained using an eye tracking system. This study was aimed at analyzing if the NSUCO test and a conventional eye tracker can detect oculomotor alterations in children with neurodevelopmental disorders and the level of correlation of the outcomes obtained with both tests.

Significant differences between CG and NDDG were found in all the categories evaluated with the NSUCO test, with worse outcomes in the group of children with neurodevelopmental disorders, as in previous studies [10,12]. Likewise, significant differences were found in some parameters provided by the eye tracking system, including the number of hypometric saccades and the percentage of hypometric saccades with respect to the number of completed saccades. This confirms that one sign characterizing the oculomotor anomaly in children with neurodevelopmental disorders is the presence of hypometric saccades. Other authors have also found alterations in saccades in children with NDD using eye tracking systems that are compatible with the results of the current series [23,24,25,26,27]. Jainta et al. [26] reported an increased saccade disconjugation in children with dyslexia, an increased disconjugate postsaccadic drift, and the uncorrelated saccade and post-saccadic drift disconjugation occurring during text reading. Furthermore, Tiadi et al. [23] demonstrated that children with dyslexia required a longer period to observe the stimulus than the control group, whereas Bucci et al. [24] found that the fixation quality and saccadic performance were poor in children with dyslexia compared to the control group. Concerning attention-deficit/hyperactivity disorder, Hakvoort Schwerdtfeger et al. [25] showed that significant saccadic regressions were present. Additionally, children with DCD have shown to experience difficulties maintaining fixation and rapid and saccadic follow-ups at a fast speed, with difficulties with saccadic inhibition and maintaining a visual goal [14]. Besides the increase in hypometric saccades in NDDG, a significantly higher percentage of regressions was also observed in this group when using a longer time interval of presentation of stimuli, 1 s. Vagge et al. [27] found significant differences between healthy and dyslexic children in the number of saccades, number of regressions, and reading time through the reading of a text. Likewise, Kraljevic and Palmovic [28] confirmed a great number of fixations, longer duration of fixations and a great number of regression saccades are the main features that differentiate the children with dyslexia form the typically developing child. This oculomotor alteration in children with NDD can have a significant impact on activities that requires an efficient use of the oculomotor system, such as reading [20,25,26] or playing sports [29,30]. Indeed, it has been suggested that visual exercises to promote oculomotor functionality may be useful to improve sports performance, although this should be investigated further [31]. 

Finally, this is the first study showing the level of correlation between the subjective evaluation of saccades with the NSUCO test and the objective measurement with the eye tracker used. Specifically, lower ability, precision and movement body/head associated NSUCO scores were found in those cases with more percentage of regressions for a time interval between stimuli of 1 s. This is a coherent finding considering that the presence of saccadic regressions is a sign of a poor ability to perform correct saccades and consequently an indicator of lower precision, with more possibility of moving head and body to compensate those errors associated to the saccadic movements. Furthermore, a higher number of saccades completed according to the eye tracking recordings was present in those cases obtaining high scores of NSUCO ability and precision for a time interval between stimuli of 0.5 and 1 s. This is also a coherent finding considering that more saccades can be completed when there is a great ability to perform precise saccadic movements. Therefore, there is a relationship between NSUCO and eye tracking outcomes when analyzing the saccadic performance in healthy children and children with NDD. More studies including larger samples sizes are needed to confirm the outcomes obtained in the current preliminary study.

This study has several limitations that should be acknowledged. First, the sample size was limited, but the objective of the current preliminary study was to detect trends to be investigated further in future clinical trials or prospective observational studies with large samples. In any case, despite the limitation of the sample size, significant differences were found between CG and NDDG in several saccadic eye movement records and significant correlations were found between subjective and objective findings. Second, although eye tracking may be considered the most adequate tool for characterizing oculomotor anomalies due to its objectivity, there is still a need for normative data and standardized diagnostic criteria using this advanced technology, allowing the clinician to differentiate in clinical practice which can be considered normal or abnormal [18]. Furthermore, a small group of children with NDD was created, but including cases with dyslexia, ADHD and DCD, with no differentiation among each neurodevelopmental condition. Future studies including a significant number of cases of each NDD should be performed to detect if there are differences among each specific type of disorder. Finally, the impact of this differential saccadic eye movement pattern in children with NDD on activities such as reading or playing sports was not specifically studied and this should be considered in future studies.

## 5. Conclusions

The presence of hypometric saccades and regressions seems to be a differential characteristic sign of children with NDD that can be detected using an objective eye tracking analysis, but also using the subjective test NSUCO that can be easily implemented in all clinical settings. This study has demonstrated for the first time that there is a significant relationship between the objective oculomotor analysis obtained with an eye tracker system and the subjective test NSUCO. Indeed, the objective eye tracker-based oculomotor parameters can be predicted from the categories evaluated with the NSUCO subjective test. This has an important clinical relevance as it confirms the validity of this fast subjective procedure to explore the oculomotricity in children with NDD and healthy children when the eye tracker technology is not available due to economic reasons or other logistic reasons. 

## Figures and Tables

**Table 1:** Scoring procedure used in the NSUCO test for the evaluation of the oculomotor function.

Performance Area	Evaluation Procedure	Scoring System
Ability	Patient’s ability of performing 5 cycles of change of fixation between the two stimuli presented	1 point: 1 cycle or no ability 2 points: 2 cycles 3 points: 3 cycles 4 points: 4 cycles 5 points: 5 cycles
Accuracy	Patient’s ability of performing 5 cycles of change of fixation without correcting refixations	1 point: significant hyper- or hypometric movements 2 points: large to moderate hyper- or hypometric movements 3 points: slight hyper or hypometric movements but constant 4 points: slight hyper or hypometric movements but intermittent 5 points: no correcting refixations
Head/body movement associated	Patient’s ability of performing 5 cycles of change of fixation without head or body movements	1 point: 1 cycle or no ability 2 points: 2 cycles 3 points: 3 cycles 4 points: 4 cycles 5 points: 5 cycles

**Table 2:** Summary of the main characteristics of the two groups involved in the study: CG, control group, and NDDG, neurodevelopmental disorder group.

Mean (SD) Median (Range)	CG (15 Children)	NDDG (17 Children)	*p*-Value
Age (years)	9.3 (1.6) 10.0 (7.0 to 12.0)	8.2 (1.4) 8.0 (6.0 to 10.0)	0.069
Sphere RE (D)	0.40 (1.30) 0.00 (0.00 to 5.00)	0.03 (0.12) 0.00 (0.00 to 0.50)	0.710
Cylinder RE (D)	−0.25 (0.78) 0.00 (−3.00 to 0.00)	−0.01 (0.06) 0.00 (−0.25 to 0.00)	0.710
Sphere LE (D)	0.43 (1.32) 0.00 (0.00 to 5.00)	0.03 (0.12) 0.00 (0.00 to 0.50)	0.710
Cylinder LE (D)	−0.23 (0.68) 0.00 (−2.50 to 0.00)	0.00 (0.00) 0.00 (0.00 to 0.00)	0.526
LogMAR CDVA RE	0.01 (0.03) 0.00 (0.00 to 0.10)	0.00 (0.00) 0.00 (0.00 to 0.00)	0.766
LogMAR CDVA LE	0.01 (0.03) 0.00 (0.00 to 0.10)	0.00 (0.00) 0.00 (0.00 to 0.00)	0.766
Phoria at distance (prism diopters)	−3.07 (3.59) −4.00 (−10.00 to 3.00)	−3.65 (4.14) −2.00 (−10.00 to 0.00)	0.823
Phoria at near (prism diopters)	−4.13 (5.21) −6.00 (−12.00 to 6.00)	−5.18 (4.95) −6.00 (−12.00 to 0.00)	0.576
NPC (cm)	7.07 (5.20) 6.00 (0.00 to 15.00)	8.12 (5.74) 8.00 (0.00 to 20.00)	0.628

SD: standard deviation, RE: right eye, LE: left eye, D: diopter, CDVA: corrected distance visual acuity, NPC: near point of convergence.

**Table 3:** Summary of the outcomes of the oculomotor examination in the two groups involved in the study: CG, control group, and NDDG, neurodevelopmental disorder group.

Mean (SD) Median (Range)	CG (15 Children)	NDDG (17 Children)	*p*-Value
	*NSUCO test*		
Saccadic Ability	4.13 (0.64) 4.00 (3.00 to 5.00)	2.12 (0.78) 2.00 (1.00 to 3.00)	<0.001
Saccadic Precision	4.27 (0.46) 4.00 (4.00 to 5.00)	2.00 (0.87) 2.00 (1.00 to 3.00)	<0.001
Body and head movements associated	4.33 (0.62) 4.00 (3.00 to 5.00)	1.53 (0.62) 1.00 (1.00 to 3.00)	<0.001
	*Eye tracker analysis*		
Number of cycles *0.5 s*	18.73 (6.65) 16.00 (13.00 to 37.00)	17.38 (4.01) 15.00 (14.00 to 25.00)	0.455
*1 s*	14.07 (1.98) 15.00 (9.00 to 16.00)	14.36 (1.34) 15.00 (11.00 to 15.00)	0.910
% of regressions *0.5 s*	31.12 (9.91) 29.00 (15.00 to 51.40)	32.19 (5.64) 31.05 (23.00 to 40.30)	0.526
*1 s*	32.26 (7.00) 31.80 (13.00 to 43.50)	37.39 (6.38) 37.50 (21.00 to 47.10)	0.012
Number of completed saccades *0.5 s*	13.80 (5.12) 15.00 (5.00 to 28.00)	11.25 (4.96) 14.00 (1.00 to 15.00)	0.089
*1 s*	10.50 (2.85) 11.00 (5.00 to 15.00)	8.71 (3.27) 8.50 (4.00 to 15.00)	0.114
% of completed saccades *0.5 s*	75.63 (21.56) 83.33 (33.33 to 100.00)	66.63 (31.35) 76.14 (6.67 to 100.00)	0.411
*1 s*	75.06 (19.35) 73.33 (45.45 to 100.00)	61.69 (24.57) 60.00 (26.67 to 100.00)	0.178
Number of hypometric saccades *0.5 s*	4.20 (2.40) 3.00 (2.00 to 10.00)	7.88 (4.88) 7.50 (2.00 to 21.00)	0.010
*1 s*	3.79 (2.97) 3.00 (0.00 to 10.00)	6.00 (2.77) 6.50 (1.00 to 10.00)	0.044
Number of hypermetric saccades *0.5 s*	1.47 (1.46) 1.00 (0.00 to 4.00)	1.69 (1.78) 1.00 (0.00 to 7.00)	0.830
*1 s*	0.57 (0.94) 0.00 (0.00 to 3.00)	1.14 (1.56) 0.50 (0.00 to 5.00)	0.401
% hypometric saccades/completed *0.5 s*	32.19 (16.81) 26.67 (13.33 to 66.67)	57.99 (30.18) 60.00 (13.00 to 100.00)	0.022
*1 s*	34.74 (21.87) 34.85 (0.00 to 71.43)	63.81 (27.07) 70.00 (7.14 to 100.00)	0.003
% hypermetric saccades/completed *0.5 s*	10.85 (11.83) 6.67 (0.00 to 40.00)	19.83 (25.04) 14.36 (0.00 to 100.00)	0.370
*1 s*	6.19 (10.14) 0.00 (0.00 to 33.33)	12.18 (14.02) 5.56 (0.00 to 33.33)	0.376

SD: standard deviation; NSUCO, Northeastern State University College of Optometry’s Oculomotor.

**Table 4:** Summary of the outcomes of the linear regression analysis for these relationships among subjective and objective oculomotor parameters showing statistically significant correlations in the whole sample (Y = objective parameter, X = subjective parameter).

Relationship	Linear Equation	R^2^	*p*-Value
Percentage regressions 1 s—Ability NSUCO	Y = 40.39 − 1.77 × X	0.096	0.108
Percentage regressions 1 s—Precision NSUCO	Y = 40.63 − 1.81 × X	0108	0.088
Percentage regressions 1 s—Head/body movement associated NSUCO	Y = 39.52 − 1.61 × X	0.118	0.073
Number saccades completed 0.5 s—Ability NSUCO	Y = 8.61 + 1.20 × X	0.083	0.109
Number saccades completed 0.5 s—Precision NSUCO	Y = 9.19 + 1.01 × X	0.069	0.146
Number saccades completed 1 s—Ability NSUCO	Y = 5.92 + 1.17 × X	0.214	0.013
Number saccades completed 1 s—Precision NSUCO	Y = 5.84 + 1.17 × X	0.230	0.010
Number hypometric saccades 0.5 s—Ability NSUCO	Y = 12.18 − 1.99 × X	0.349	<0.001
Number hypometric saccades 0.5 s—Precision NSUCO	Y = 11.09 − 1.63 × X	0.274	0.002
Number hypometric saccades 0.5 s—Head/body movement associated NSUCO	Y = 9.29 − 1.13 × X	0.173	0.018
Percentage hypometric saccades 0.5 s—Ability NSUCO	Y = 84.81 − 12.71 × X	0.324	0.001
Percentage hypometric saccades 0.5 s—Precision NSUCO	Y = 79.15 − 10.86 × X	0.276	0.002
Percentage hypometric saccades 0.5 s—Head/body movement associated NSUCO	Y = 69.61 − 8.34 × X	0.217	0.007
Percentage hypometric saccades 1.0 s—Ability NSUCO	Y = 82.25 − 10.49 × X	0.211	0.014
Percentage hypometric saccades 1.0 s—Precision NSUCO	Y = 86.71 − 11.65 × X	0.280	0.004
Percentage hypometric saccades 1.0 s—Head/body movement associated NSUCO	Y = 74.94 − 8.76 × X	0.219	0.012

## Data Availability

Data available on request due to privacy/ethical restrictions.
